# Centromere-associated repeat arrays on *Trypanosoma brucei *chromosomes are much more extensive than predicted

**DOI:** 10.1186/1471-2164-13-29

**Published:** 2012-01-18

**Authors:** Maria C Echeverry, Christopher Bot, Samson O Obado, Martin C Taylor, John M Kelly

**Affiliations:** 1 Department of Infectious and Tropical Diseases, London School of Hygiene and Tropical Medicine, Keppel Street, London WC1E 7HT, UK; 2 Laboratorio de Parasitologia - Facultad de Medicina, Universidad Nacional de Colombia-Sede, Bogota, Columbia; 3 Laboratory of Cellular and Structural Biology, Rockefeller University, 1230 York Avenue, New York, NY 10065, USA

## Abstract

**Background:**

African trypanosomes belong to a eukaryotic lineage which displays many unusual genetic features. The mechanisms of chromosome segregation in these diploid protozoan parasites are poorly understood. Centromeres in *Trypanosoma brucei *have been localised to chromosomal regions that contain an array of ~147 bp AT-rich tandem repeats. Initial estimates from the genome sequencing project suggested that these arrays ranged from 2 - 8 kb. In this paper, we show that the centromeric repeat regions are much more extensive.

**Results:**

We used a long-range restriction endonuclease mapping approach to more accurately define the sizes of the centromeric repeat arrays on the 8 *T. brucei *chromosomes where unambiguous assembly data were available. The results indicate that the sizes of the arrays on different chromosomes vary from 20 to 120 kb. In addition, we found instances of length heterogeneity between chromosome homologues. For example, values of 20 and 65 kb were obtained for the arrays on chromosome 1, and 50 and 75 kb for chromosome 5.

**Conclusions:**

Our results show that centromeric repeat arrays on *T. brucei *chromosomes are more similar in size to those of higher eukaryotes than previously suspected. This information provides a firmer framework for investigating aspects of chromosome segregation and will allow epigenetic features associated with the process to be more accurately mapped.

## Background

Centromeres are the chromosomal loci that facilitate segregation in most eukaryotes. They are the site of assembly of the kinetochore, the nucleoprotein complex which anchors the microtubule spindles that separate sister chromatids and mediate their movement to the daughter nuclei. Most centromeres are "regional" and encompass large sections of DNA, spanning 0.06 - 5 Mb, in species as diverse as plants, insects and mammals [1-3]. Centromeric DNA is typically comprised of arrays of highly repeated sequences, interrupted by transposable elements [4,5]. The repeats are generally restricted to centromeric regions and are often in the size range 150 - 180 bp. This length is similar to that of nucleosomes, a property that may be of functional significance [6]. Although many features of centromeric DNA are widespread, there is little sequence conservation, even between closely related species [7], and most evidence suggests that centromeres are determined epigenetically [8,9].

In human chromosomes, centromeres have a conserved core of α-satellite repeats (~170 bp) stretching over several megabases, which is flanked by extensive regions that contain multiple retrotransposon insertions [4]. In eukaryotic microorganisms, centromeres can also encompass large regions of chromosomal DNA. Those of *Schizosaccharomyces pombe *for example, range from 35 - 110 kb [10] and are organised as chromosome-specific core elements, flanked by inverted arrays of 3 - 7 kb. These in turn are flanked by more extensive outer repeats. Unusually in *Saccharomyces cerevisiae*, the regions that specify kinetochore assembly are restricted to single 125 bp elements termed "point" centromeres [11]. Some organisms, such as *Caenorhabditis elegans*, have holocentric chromosomes that lack specific centromeres [12]. In these instances, microtubules bind along the entire length of the chromosome.

Protozoan parasites of the *Trypanosoma brucei *species complex are insect-transmitted pathogens that are of major medical and veterinary importance throughout sub-Saharan Africa. They belong to the Excavata, a eukaryotic lineage which includes the other trypanosomatid parasites *Trypanosoma cruzi *and *Leishmania *species. Several features of gene organisation and expression in these organisms are unusual. Protein coding genes lack conventional RNA polymerase II (pol II) promoters [13] and are organised in long co-directional clusters which can stretch for tens to hundreds of kilobases [14]. Transcription is polycistronic, and processing involves a *trans*-splicing mechanism in which all mRNAs are modified post-transcriptionally by the addition of a 39-nucleotide spliced leader to their 5'-ends. *T. brucei *has a haploid genome content of 35 Mb, with 11 megabase pair chromosomes (0.9 - 5.7 Mb). Unusually, chromosome homologues can vary significantly in size [14]. In addition, this parasite also contains two classes of atypical nuclear chromosomes; the intermediate-size chromosomes (300 - 900 kb) that contain some variant surface glycoprotein (*VSG) *genes, but no house-keeping genes, and the minichromosomes (50 - 100 kb), which appear to act as a reservoir of *VSG *sequences [15].

The *T. brucei *genome project was completed in 2005 [14]. However, sequence elements characteristic of centromeric DNA in other eukaryotes were not described. Furthermore, candidates for the 'core' centromeric proteins and most of the other factors involved in kinetochore assembly could not be identified [14,16]. This includes the variant histone CenH3, which specifies centromere location in eukaryotes and was thought to be ubiquitous [17]. The first evidence on the nature and location of centromeric DNA in *T. brucei *came from a biochemical mapping approach based on etoposide-mediated topoisomerase-II cleavage [18,19]. Topoisomerase-II has a major regulatory role in chromosome segregation and accumulates at centromeres during late metaphase, where it resolves the catenated DNA strands that provide the final structural link between sister kinetochores [20,21]. This process requires double stranded DNA cleavage, passage of the uncut duplex through the gap and re-ligation to repair the break. Etoposide inhibits this re-ligation step leading to lesions in chromosomal DNA at sites of topoisomerase-II activity. In human chromosomes, etoposide-mediated cleavage sites occur within the α-satellite repeats that constitute centromeric DNA [22,23]. In both *T. cruzi *[24] and *Plasmodium *[25,26], these sites have been delineated to chromosomal loci that confer mitotic stability. In *Toxoplasma gondii*, they co-locate with the binding sites of the centromeric histone CenH3 [27].

Using the etoposide mapping method, we identified the location of putative centromeric domains on the 8 *T. brucei *chromosomes that had been fully assembled [18]. These loci, which occur once per chromosome, encompass regions between directional gene clusters that contain transposable elements and an array of AT-rich repeats predicted to extend between 2 and 8 kb. The tandem repeats are arranged in units of ~147 bp and share intra-chromosomal identities ranging from 50% to more than 90%. The units have a complex structure made up of degenerate sub-repeats of ~48 and ~30 bp (for a more detailed description of their make-up, see reference [18]). We also noted that the repeat arrays were located adjacent to ribosomal RNA genes on 5 of the chromosomes, although the significance of this is unknown. The intermediate and minichromosomes did not exhibit site-specific topoisomerase-II activity, suggesting that their segregation might involve a centromere-independent mechanism, a finding consistent with the "lateral-stacking" model [15].

In the initial analysis of the *T. brucei *centromeric domains, we identified discrepancies between the published sequence data of two chromosomes and our preliminary long range restriction mapping [18]. We also found evidence of heterogeneity in the extent of these regions between chromosome homologues. However, it was unclear whether the differences arose from an under-estimation of the copy number of the tandem repeats, whether they were due to the gaps in the assembly of the adjacent regions, or whether this under-estimation of size was also the case with other *T. brucei *chromosomes. Here, we show that the centromeric repeats in *T. brucei *chromosomes are present at much higher copy number than predicted, with an organisation that is more typical of centromeric domains in higher eukaryotes than realised. These data provide a more complete model for *T. brucei *chromosome structure, an improved basis for investigating the mechanisms of segregation, and will enable more detailed functional mapping of this crucial chromosomal region to be undertaken.

## Methods

### Parasites and DNA preparation

*T. brucei *procyclic forms (genome project strain TREU 927/4) were grown in SDM-79 medium [28] with 10% heat-inactivated fetal bovine serum at 28°C. For preparation of intact chromosomal DNA, the agarose embedding technique was used [29]. 10^8 ^procyclics were immobilized in 1% low melting-point agarose blocks and incubated at 48°C for 48 hours in proteinase K/sarcosyl buffer. Genomic DNA was extracted using the phenol-chloroform method [30].

### In situ digestion and electrophoretic resolution

Prior to incubation with restriction endonucleases, agarose blocks were washed 3 times for 1 hour at 48°C in 50 volumes of TE buffer (10 mM Tris-HCl, 1 mM EDTA, pH 7.4) containing 40 μg ml^-1 ^phenylmethanesulphonylfluoride to inactivate proteinase K. After a minimum of 2 hours equilibration with the respective restriction enzyme buffer, blocks were incubated with restriction enzymes for 48 hours at 37°C. Fresh enzyme (5 - 10 units) was added at the 24 and 36 hour time points. The digested DNA was resolved by a CHEF (contour-clamped homogenous electric field) Mapper System (Bio-Rad) (typically quarter of a block was used per lane) using an auto-algorithm set to the designated molecular mass range. For resolution of DNA fragments less than 20 kb, genomic DNA (5 - 10 μg) was digested for 3 hours and fractionated on 0.5% agarose gels using standard electrophoresis techniques. As molecular size markers, a combination of Bio-Rad CHEF DNA standards 8 - 48 kb, lambda ladder 50 - 1000 kb and *S. cerevisiae *chromosomes from 225 - 2,200 kb were used. Southern blotting was performed using standard procedures as outlined previously [24].

## Results

The locations of centromeric regions on *T. brucei *chromosomes 9 - 11 have been predicted from etoposide-mediated mapping [19], however incomplete assembly of the corresponding regions negates accurate long-range restriction mapping. For this study, we therefore focused on *T. brucei *chromosomes 1 - 8 (Table 1). As a first step, we generated *in silico *restriction digestion maps, based on the sequences available in GeneDB (Additional file 1). Our aim was to identify enzymes which cut proximal to the ends of centromeric arrays [31] and allowed the generation of paired overlapping fragments containing the repeat arrays, which could then be sized following electrophoresis. We also sought to identify sequences located adjacent to the centromeric region to act as single copy probes. Mostly these were open reading frames (Additional file 1). The abundance of high copy number elements adjacent to the tandem repeats (typically retrotransposons and ribosomal RNA genes) was in some cases a limiting factor. Below we describe our approach to delineating the repeat arrays, using chromosomes 3, 5 and 7 as examples. The complete data set, including full analysis of the other chromosomes, is shown in Additional files 2 and 3, and summarised in Table 1. Fragment sizes greater than 20 kb were estimated to the nearest 5 kb.

**Table 1:** Inferred sizes of the centromeric tandem arrays on chromosomes 1 - 8.

**Chr no**.	Chr size	Size of centromeric repeat array (GeneDB)	Size inferred^a^ from mapping	Artemis coordinates^b ^
**1**	1.15/1.2 Mb	5.2 kb	20 & 65 kb	780179....785416
**2**	1.3 Mb	8.2 kb	30 & 55 kb	290326....298494
**3**	1.8/2.0 Mb	7.8 kb	75 & 80 kb	884223....891983
**4**	1.9/2.0 Mb	3.6 kb	70 kb	954159....957772
**5**	2.0 Mb	1.9 kb	50 & 75 kb	197919....199815
**6**	2.0/2.5 Mb	5.4 kb	55 kb	59066....64433
**7**	2.7 Mb	3.1 kb	100 & 120 kb	1936979....1940105
**8**	2.7/2.9 Mb	unknown**^c^**	100 kb	2233201....2233419**^d^**

**^a^**The approaches used to infer the extent of each of the centromeric repeat arrays are outlined in the text (chromosomes 3, 5 and 7) and Additional file 2 (chromosomes 1, 2, 4, 6 and 8).

**^b^**The coordinates were defined using the Tandem Repeats Finder Program [31]. The positions in the sequence where the programme detected the first and the last motif were defined as the beginning and as the end of the centromeric repeat region. Coordinates are from *T. brucei *927 version 4 (GeneDB).

**^c^**The size of the centromeric repeat array is not defined in GeneDB due to incomplete gap closure.

**^d^**The coordinates correspond to blast hits with chromosome 4 centromeric repeat sequences. These occur adjacent to a gap of undetermined size.

### Chromosome 3

**Figure 1:**
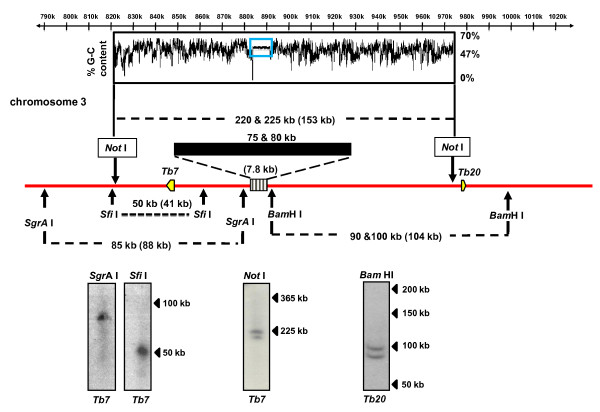
**Delineating the centromeric repeats on *T. brucei *chromosome 3 using long range restriction mapping**. Chromosomal DNA was immobilised in agar blocks, restriction digested and fractionated by CHEFE (Methods). Blots were hybridised using the probes *Tb7 *and *Tb20 *(Additional file 1) (identified as yellow arrows, which also signify the direction of transcription). Fragment sizes derived in this study are shown on the schematic, with their predicted sizes (GeneDB) in parentheses. The location and inferred size of the ~147 bp repeat array (7.8 kb, GeneDB; 75/80 kb, this work) is shown (striped/filled box). The %GC content across the region is presented using Artemis [44], with the location of the repeat arrays shown in a blue box.

Sequence data (GeneDB) had suggested that the repeat array on this chromosome could be isolated on a *Not *I fragment of 153 kb. However, Southern analysis of *Not *I digested DNA, following fractionation by CHEF (Figure 1), revealed fragments of 220 and 225 kb. This indicated the presence of ~70 kb of additional DNA in this region and was consistent with heterogeneity between chromosome homologues. Two enzymes, *Sgr*A I and *Sfi *I, were used for analysis of sequences upstream of the array. Single fragments were identified, which were in both cases slightly larger (5/10 kb) than predicted. To investigate the downstream region, we used *Bam*H I, which cuts within 2 kb of the repeat array and was predicted to liberate a fragment of 104 kb. This produced two fragments on the autoradiograph, one of which was 10 - 15 kb shorter than expected. These small differences, which may arise from the haploid mosaic nature of the genome sequence [14], cannot account for the larger than predicted size of the *Not *I fragment. The data therefore suggest that the centromeric repeat region on this chromosome is considerably more extensive than expected and that it may be of slightly different length on each homologue. This latter inference is not unambiguous, because of the heterogeneity in the flanking *Bam*H I fragment. A paucity of single copy probes and convenient restriction sites in this region limited our ability to address this further.

### Chromosome 5

**Figure 2:**
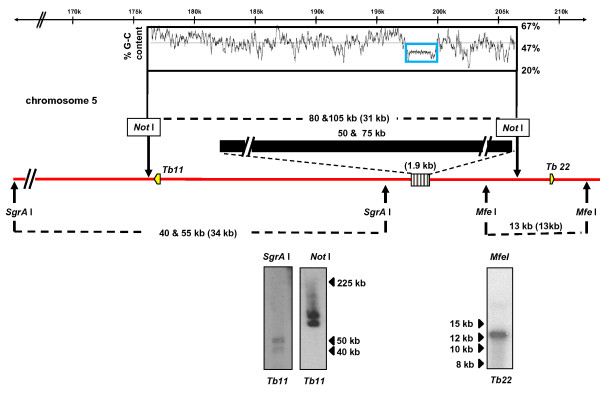
**The extent of the centromeric tandem repeats on *T. brucei *chromosome 5**. Long range restriction mapping was carried out as described previously (Methods, Figure 1) using probes *Tb11 *and *Tb22 *(Additional file 1). Fragment sizes derived in this study are indicated on the schematic, with the values from GeneDB in parentheses.

In accordance with genome sequence data, digestion with *Not *I should have generated a 31 kb fragment that contains the centromeric repeat array from chromosome 5. However, Southern hybridisation identified fragments of 80 and 105 kb (Figure 2), implying heterogeneity between homologues and the presence of 50 and 75 kb of additional DNA in the centromeric region. Analysis of an *Mfe *I digest (Figure 2) indicated that this did not arise from additional sequences in the immediate downstream region. When the upstream region was analysed following an *Sgr*A I digest, two fragments were identified, which were 5 and 20 kb larger than predicted. Therefore, some of the size heterogeneity observed with the *Not *I digest could be due to additional sequences in this upstream region. However, the vast majority of the additional sequence in the *Not *I fragment, must arise from DNA within, or immediately adjacent to the repeat array (Figure 2).

### Chromosome 7

**Figure 3:**
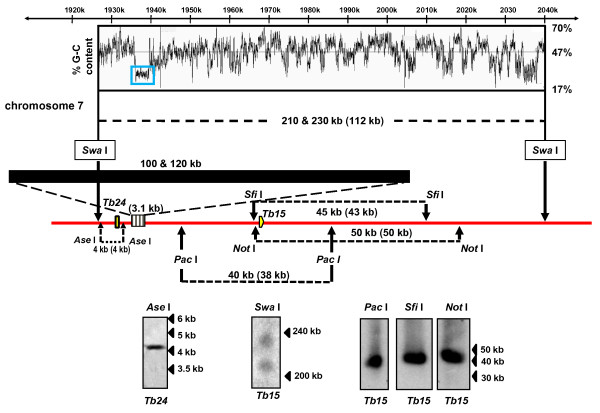
**The extent of the centromeric tandem repeats on *T. brucei *chromosome 7**. Analysis was carried out as described for Figures 1 and 2 using probes *Tb24 *and *Tb15 *(Additional file 1). Probe *Tb24 *was derived from a unique intergenic sequence. Fragment sizes derived in this study are indicated on the schematic, with the values from GeneDB in parentheses. The data suggest that the repeat arrays vary in length between chromosome homologues and stretch over at least100 kb (see also Table 1).

The repeat array on this chromosome was predicted to be located on a *Swa *I fragment of 112 kb. Southern analysis however, identified a doublet of 210 and 230 kb (Figure 3). Digestion with *Ase *I demonstrated that this was not due to any additional sequences in the immediate upstream region. Likewise, analysis of *Pac *I, *Sfi *I and *Not *I digests (Figure 3) identified downstream fragments similar to the sizes predicted on GeneDB. These data are consistent the heterogeneity between chromosomes, with the repeat arrays stretching over approximately 100 kb and 120 kb.

## Discussion and Conclusions

In eukaryotes, centromeric sequences are frequently organised as highly repetitive tandem arrays that stretch over extensive regions of chromosomal DNA. Their complete assembly has proven to be an intractable problem in most genome projects [32]. Where detailed analysis has been undertaken, considerable intra-chromosomal size variation and sequence divergence has become apparent [3,33]. This arises from the acquisition of point mutations and high rates of unequal homologous recombination. When the *T. brucei *genome was initially completed [14], regions subsequently identified as centromeres, were characterised by the presence of ~147 bp repeat arrays predicted to extend over 2 - 8 kb [18]. In each of the 8 *T. brucei *chromosomes analysed here, our data suggest that these centromeric arrays are much larger (Table 1), varying from 20 kb (chromosome 1) to more than 100 kb (chromosome 7). We found a tendency for these regions to be more extensive in the larger chromosomes. This contrasts with *S. pombe*, where centromere length is inversely proportional to the chromosome length [34]. In addition, we also observed several instances of heterogeneity between chromosome homologues (Table 1). Using data available on TriTrypDB, there is no evidence for single nucleotide polymorphisms contributing to the generation of larger than expected restriction fragments containing the arrays. Although we cannot demonstrate unambiguously that the missing segments of centromeric DNA are constituted by tandem repeats, it would be unusual if extensive segments of non-repetitive sequences had been missed from the corresponding regions of each chromosome during the genome project.

The nature of the centromere-kinetochore complex in trypanosomes and the role that the ~147 bp repeats play in recruitment are two of the most intriguing unsolved questions in parasite biology. Although segregation in trypanosomes appears to be mediated by a conventional microtubule - kinetochore attachment, the number of kinetochores seems to be less than the number of chromosomes [35,36]. Trypanosomatids lack genes for the conserved "core" centromeric proteins, as well as the majority of other proteins involved in kinetochore assembly and function [14]. They are distinct in lacking an obvious orthologue of the variant histone CenH3, which replaces the canonical histone H3 at centromeres in other eukaryotes. In *T. brucei*, the only histone H3 variant identified is non-essential, enriched at telomeres, and lacks the extended loop I region or characteristic carboxyl terminal domain that are diagnostic of CenH3 [37-39] .

In other eukaryotes, CenH3 is essential for kinetochore assembly and functions as an epigenetic marker for centromere location [40]. By contrast, the centromeric repeat arrays with which they interact are not a pre-requisite. If normal centromere function is lost in some eukaryotes, neocentromeres can form in regions which lack these arrays, and once formed, the new location is stably inherited and specified epigentically by CenH3 binding [3,40]. Centromeric repeats then accumulate in these regions over time, where they may have a role in providing an environment that favours or promotes the formation of centromeric chromatin. Traditionally, centromeric heterochromatin had been considered transcriptionally quiescent. However several recent studies, initially in fission yeast, have highlighted an essential role for short interfering RNAs (siRNAs) derived from centromeric sequences in the formation of heterochromatin and centromere function [41,42]. Interestingly, a recent report has described a class of siRNAs derived from centromeric repeats in *T. brucei*, although their functional significance remains to be elucidated [43].

Our finding that the ~147 bp tandem repeats constitute a larger than expected component of *T. brucei *chromosomes provides an improved framework for investigating aspects of genome biology, including the determinants of centromere function. For example, the cell-cycle specific accumulation of topoisomerase-II at centromeres is required for regulated segregation of sister chromatids. Precise mapping of this decatenation activity onto *T. brucei *chromosomes was complicated by uncertainty over the size of the centromeric repeats arrays [18]. Likewise, analysis of chromatin immunoprecipitation experiments to assess the extent of histone modifications associated with centromeric domains would be difficult to interpret in the absence of a more accurate chromosome map. To date most studies on chromosome segregation have focused on mammals, insects, plants and fungi. Analysis of the situation in trypanosomes demonstrates both similarities and differences from the standard model. We have now shown that the organisation of centromeric DNA repeats in *T. brucei *conforms to the "regional" class, typical of higher eukaryotes. In contrast, the protein factors which mediate segregation are unknown, and by inference, must be highly divergent. Further studies aimed at uncovering the mechanisms involved are crucial to ensure that our understanding the chromosome segregation takes full account of eukaryotic diversity.

## List of Abbreviations

CenH3: centromeric histone H3; CHEF(E): contour-clamped homogenous electric field (electrophoresis); siRNA, short interfering RNA; VSG: variant surface glycoprotein.

## Authors' contributions

MCE, designed the study, carried out the experiments and wrote the paper. CB and SOO made the original observations, contributed to experimental design and commented on the manuscript. MCT contributed to experimental design and commented on the manuscript. JMK designed the study and wrote the paper. All authors read and approved the final manuscript.

## Supplementary Material

Additional file 1 **Details of probes and restriction sites used for mapping**. A comprehensive list of DNA probes used for restriction mapping and the Artemis coordinates of the corresponding restriction enzymes. Click here for file

Additional file 2 **Analysis of restriction endonuclease mapping data for *T. brucei *chromosomes 1, 2, 4, 6, and 8**. Details on how the mapping data for those chromosomes not described in the text of the manuscript were interpreted. Click here for file

Additional file 3 **Delineation of the centromeric repeats on *T. brucei *chromosomes 1 - 8 using long range restriction mapping**. A complete collation of the mapping data from all of the *T. brucei *chromosomes analysed. Click here for file
