# Supplementary material for: Centromere-associated repeat arrays on Trypanosoma brucei chromosomes are much more extensive than predicted
Source: BMC Genomics. 2012 Jan 18;13:29. doi: 10.1186/1471-2164-13-29 (PMC3292466; doi:10.1186/1471-2164-13-29)
Supplement: Additional file 1 — Details of probes and restriction sites used for mapping. A comprehensive list of DNA probes used for restriction mapping and the Artemis coordinates of the corresponding restriction enzymes. [file 1471-2164-13-29-S1.DOC]

**Additional File 1. Details of probes and restriction sites used for mapping**

| **Chromosome** | **Probes used for mapping** | **Artemis coordinates of the restriction sites used.** |
| --- | --- | --- |
| **Chr 1** (Tb927_**01**_v4) | Tb2-Tb927.1.3560 (758686..761285)  Tb3-Tb927.1.3830 (816420..818243) | *Not* I: 713357..832852  *Sfi* I: 789136..841227  *Sma* I: 758739..775468 |
| **Chr 2** (Tb927_**02**_v4) | Tb5 - Tb927.2.1380 (254762..257272)  Tb19 - Tb927.2.1700 (317225..319513) | *Swa* I: 243222..344860  *Cla* I: 232788..270963  306970..329391  *Bam*H I: 227490..258552  300054..326600 |
| **Chr 3** (Tb927_**03**_v4) | Tb7 - Tb927.3.3300 (848040.. 850484)  Tb20 - Tb927.3.3440 (977889..978926) | *Not* I: 821575..974940  *Sfi* I: 820941..861808  *Bam*H I: 893667..997799  *Sgr*A I: 791545..879300 |
| **Chr 4** (Tb927_**04**_v4) | Tb9 - Tb927.4.3690 (932834..934153)  Tb21 - Tb927.4.3760 (981586..982791) | *Not* I: 929645..1023706  *Cla* I: 961488..989023  *Bam*H I: 976765..992174  *Sma* I: 932277..948871 |
| **Chr 5** (Tb927_**05**_v4) | Tb11 - Tb927.5.570 (175655..182038)  Tb22 - Tb927.5.630 (209175..210380) | *Not* I: 175963..206764  *Sgr*A I: 161688..195954  *Mfe* I: 204160..217163 |
| **Chr 6** (Tb927_**06**_v4) | Tb23 - intergenic (107870..108350)  Tb25 - intergenic (38968..39976) | *Pac* I: 18830..271659  *Sfi* I: 66533..233873 |
| **Chr 7** (Tb927_**07**_v4) | Tb15 - Tb927.7.6910 (1967785..1970257)  Tb24 - intergenic (1932463..1933183) | *Swa* I: 1928566..2040733  *Sfi* I: 1967865..2010430  *Pac* I: 1947780..1985472  *Not* I: 1967927..2018204  *Ase* I: 1929732..1933852 |
| **Chr 8** (Tb927_**08**_v4) | Tb 16 - Tb927.8.7710 (2221274..2221933)  Tb 17 - Tb927.8.7770 (2256953..2258632) | *Not* I: 2218956..2273363  *Sex*A I: 2221682..2228453  *Mfe* I: 2216728..2221342  *Mlu* I: 2242263..2259971 |
