# Supplementary material for: Centromere-associated repeat arrays on Trypanosoma brucei chromosomes are much more extensive than predicted
Source: BMC Genomics. 2012 Jan 18;13:29. doi: 10.1186/1471-2164-13-29 (PMC3292466; doi:10.1186/1471-2164-13-29)
Supplement: Additional file 2 — Analysis of restriction endonuclease mapping data for T. brucei chromosomes 1, 2, 4, 6, and 8. Details on how the mapping data for those chromosomes not described in the text of the manuscript were interpreted. [file 1471-2164-13-29-S2.DOC]

**Additional file 2. Analysis of restriction endonuclease mapping data for *T. brucei* chromosomes 1, 2, 4, 6, and 8.** (See additional file 3 for further details)

***Chromosome 1***

According to data obtained from GeneDB, *Not* I digestion of this chromosome should release a fragment of 120 kb containing the centromeric repeat array and associated flanking sequences. However, when assessed by Southern hybridization, a doublet of 140 and 185 kb was observed (Additional file 3A). To determine whether this size discrepancy arose from an underestimation of the extent of tandem repeats, *Sma* I and *Sfi* I digestions were performed to delineate fragments immediately upstream and downstream. *Sma* I yielded a fragment of 17 kb, the expected size. *Sfi* I digestion generated a doublet of 40 and 50 kb, as opposed to the predicted single band of 52 kb. The centromeric repeats are therefore considerably larger than predicted and vary in size between homologues. Lengths of 20 and 65 kb can be inferred, although due to the heterogeneity of the *Sfi* I products, one of the centromeric arrays may be 10 kb larger.

***Chromosome 2***

Digestion with *Swa* I was predicted to generate a fragment of 102 kb containing the centromeric repeats and associated flanking regions (Additional file 3B). However, Southern blotting identified a doublet of 130 and 155 kb. Analysis of the upstream and downstream flanking DNA, using independent single digestion with *Cla* I or *Bam*H I, demonstrated that the additional sequences were not located in these regions. Indeed, our data indicate that the upstream *Cla* I fragment derived from one of the chromosome homologues is ~20 kb smaller than expected. The most likely inference therefore, is that the centromeric repeats in this chromosome approximate to at least 30 and 55 kb, as opposed to the previously assumed value of 8 kb. One of the arrays though, may be 20 kb larger than stated, since the estimate is based on the *Cla* I fragment being the same size (40 kb) on both homologues.

***Chromosome 4***

*Not* I digestion was expected to release a 94 kb fragment incorporating the repeat array. On Southern blots, we found that the corresponding fragment migrates as a 165 kb band (Additional file 3D). A search for single nucleotide polymorphisms within strain 927 (TriTrypDB) identified a site within one of the *Not* I recognition sequences (position 929,643) that generated this fragment. However, this could not account for the size of the fragment generated, since the proximal upstream *Not* I site is only 17 kb distant (position 912,303). Analysis of the upstream region using *Sma* I digestion, identified a 20 kb fragment, marginally larger than predicted. When the downstream flanking region was assessed using *Cla* I and *Bam*H I, the fragments generated were also in the predicted range. Therefore, we estimate that the centromeric repeats in this chromosome encompass up to 70 kb.

***Chromosome 6***

The chromosome 6 centromeric repeat array is located in the vicinity of the sub-telomeric region. This limited analysis of the upstream flanking sequences (Additional file 3F). However, we did identify a *Sfi* I restriction site 2.1 kb downstream of the array, which was ideally place for investigating the size of the downstream region. On Southern blots probed with a unique intergenic sequence (Additional file 1), a 160 kb fragment was observed, similar to the size expected (Additional file 3F). After probing a *Pac* I digest, which releases a fragment encompassing the centromeric repeats and their flanking regions, we detected a band of 315 kb, as opposed to the 253 kb band predicted. Taken together, these data suggest that the centromeric repeats stretch for at least 55 kb.

***Chromosome 8***

The tandem repeat array for chromosome 8 is located in an unclosed region rich in RIME and INGI elements, predicted to be released as a fragment of 54 kb following *Not* I digestion (Additional file 3H). When we determined the size experimentally, we detected a fragment of 155 kb. The upstream array flanking region was mapped using independent digestions with *Mfe* I and *Sex*A I. This yielded the expected 5 kb and 7 kb fragments. The downstream flanking region was analysed using *Mlu* I. This produced a fragment of 17 kb, also of the predicted size. This suggests that the unclosed region corresponding to the centromeric repeats should be about 100 kb. The arrows adjacent to the unresolved repeat region in additional file 3G (Artemis coordinates: 2236404..2237074) correspond to INGI (Artemis coordinates: 237286..2238689) and RIME-A (Artemis coordinates: 2239214..2239407) retroposon–like elements.
