# Supplementary material for: Centromere-associated repeat arrays on Trypanosoma brucei chromosomes are much more extensive than predicted
Source: BMC Genomics. 2012 Jan 18;13:29. doi: 10.1186/1471-2164-13-29 (PMC3292466; doi:10.1186/1471-2164-13-29)
Supplement: Additional file 3 — Delineation of the centromeric repeats on T. brucei chromosomes 1 - 8 using long range restriction mapping. A complete collation of the mapping data from all of the T. brucei chromosomes analysed. [file 1471-2164-13-29-S3.PPT]

## Slide 1
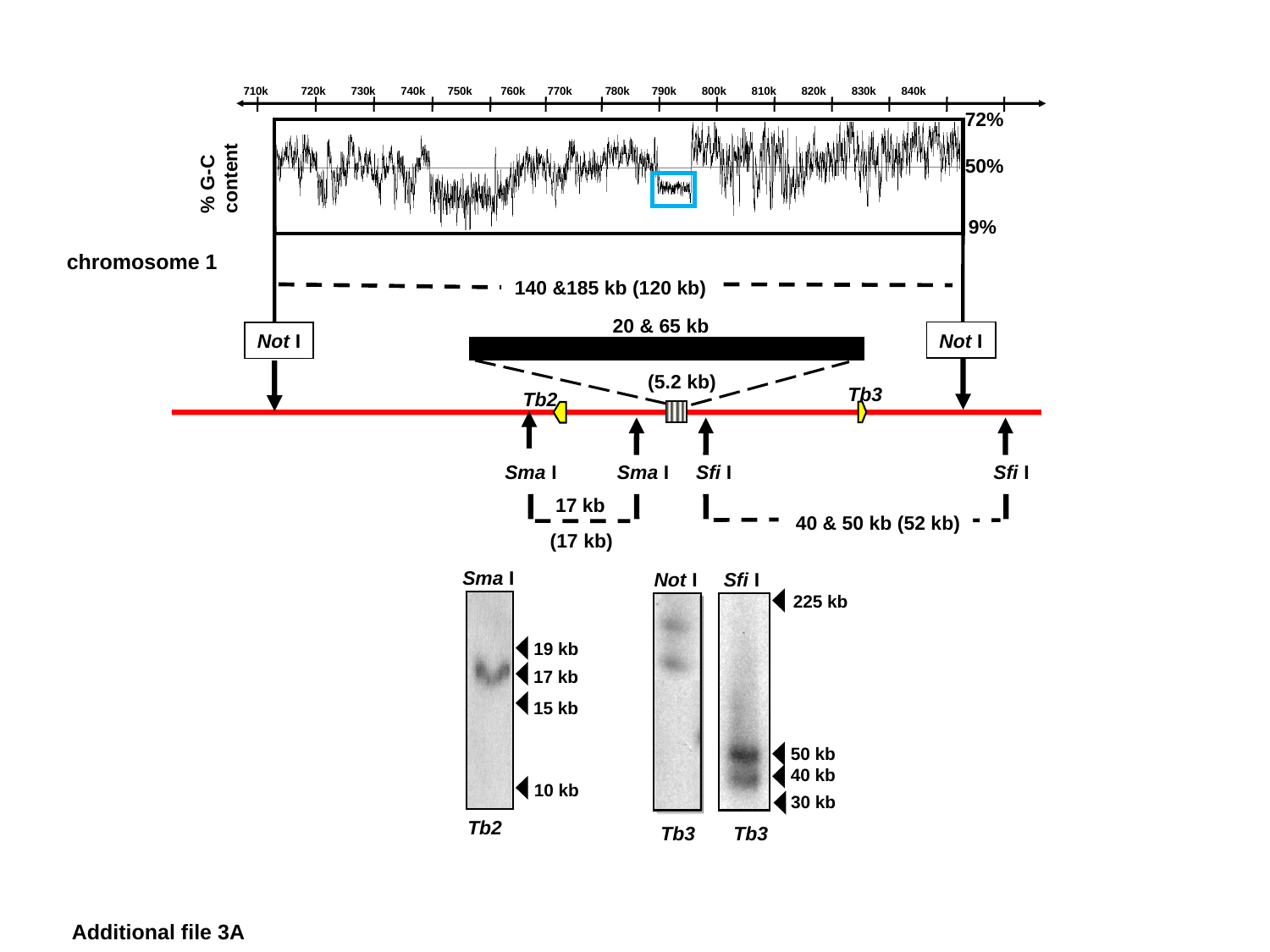

710k 720k 730k 740k 750k 760k 770k 780k 790k 800k 810k 820k 830k 840k
72%
50%
% G-C
content
9%
chromosome 1
140 &185 kb (120 kb)
20 & 65 kb
Not I
Not I
(5.2 kb)
Tb3
Tb2
Sfi I
Sfi I
Sma I
Sma I
 17 kb
 (17 kb)
40 & 50 kb (52 kb)
Sma I
Sfi I
Not I
225 kb
19 kb
17 kb
15 kb
10 kb
50 kb
40 kb
30 kb
Tb2
Tb3 Tb3
Additional file 3A
~22025kb

## Slide 2
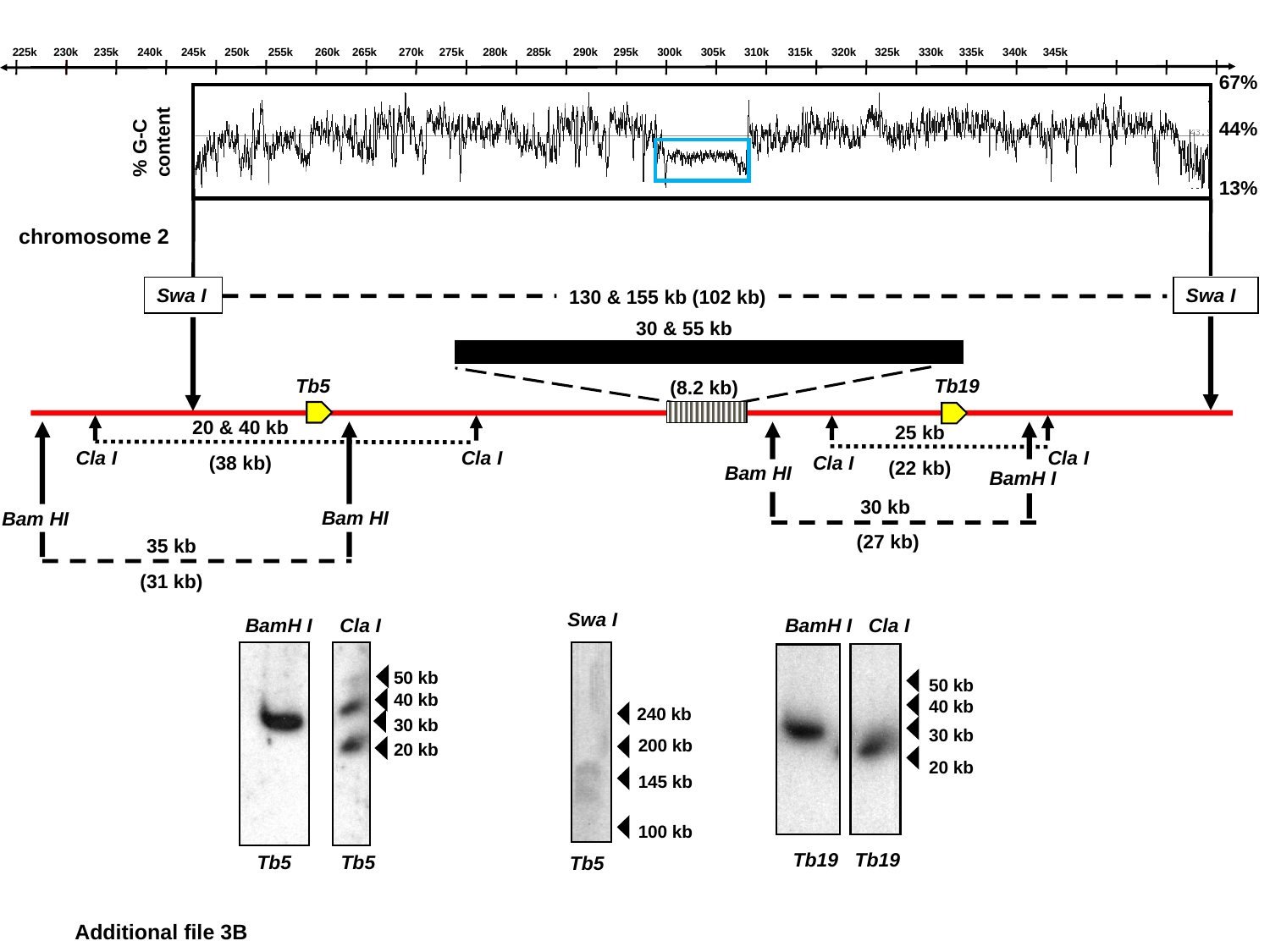

225k 230k 235k 240k 245k 250k 255k 260k 265k 270k 275k 280k 285k 290k 295k 300k 305k 310k 315k 320k 325k 330k 335k 340k 345k
67%
44%
% G-C
content
13%
chromosome 2
Swa I
Swa I
130 & 155 kb (102 kb)
30 & 55 kb
Tb5
Tb19
(8.2 kb)
20 & 40 kb
(38 kb)
25 kb
(22 kb)
Cla I
Cla I
Cla I
Cla I
Bam HI
BamH I
30 kb
 (27 kb)
Bam HI
Bam HI
35 kb
(31 kb)
Swa I
BamH I Cla I
BamH I Cla I
50 kb
50 kb
40 kb
40 kb
240 kb
30 kb
30 kb
20 kb
200 kb
20 kb
145 kb
100 kb
Tb19 Tb19
 Tb5 Tb5
Tb5
Additional file 3B

## Slide 3
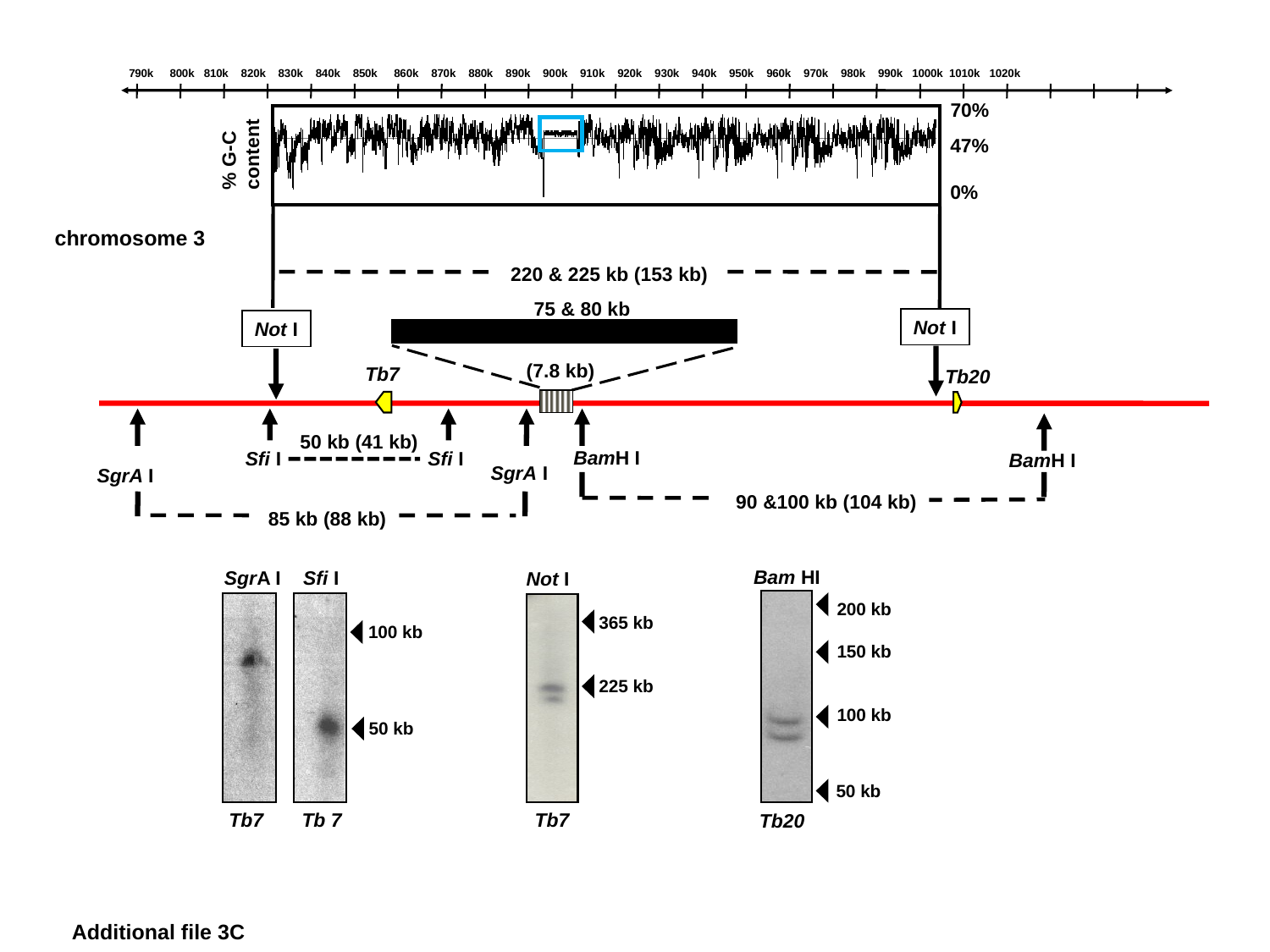

790k 800k 810k 820k 830k 840k 850k 860k 870k 880k 890k 900k 910k 920k 930k 940k 950k 960k 970k 980k 990k 1000k 1010k 1020k
47%
0%
70%
% G-C
content
chromosome 3
220 & 225 kb (153 kb)
75 & 80 kb
Not I
Not I
(7.8 kb)
Tb7
Tb20
50 kb (41 kb)
BamH I
Sfi I
Sfi I
 BamH I
SgrA I
SgrA I
90 &100 kb (104 kb)
85 kb (88 kb)
Bam HI
 SgrA I Sfi I
Not I
200 kb
150 kb
100 kb
365 kb
225 kb
100 kb
50 kb
50 kb
Tb20
Tb7 Tb 7
Tb7
Additional file 3C
~22025kb

## Slide 4
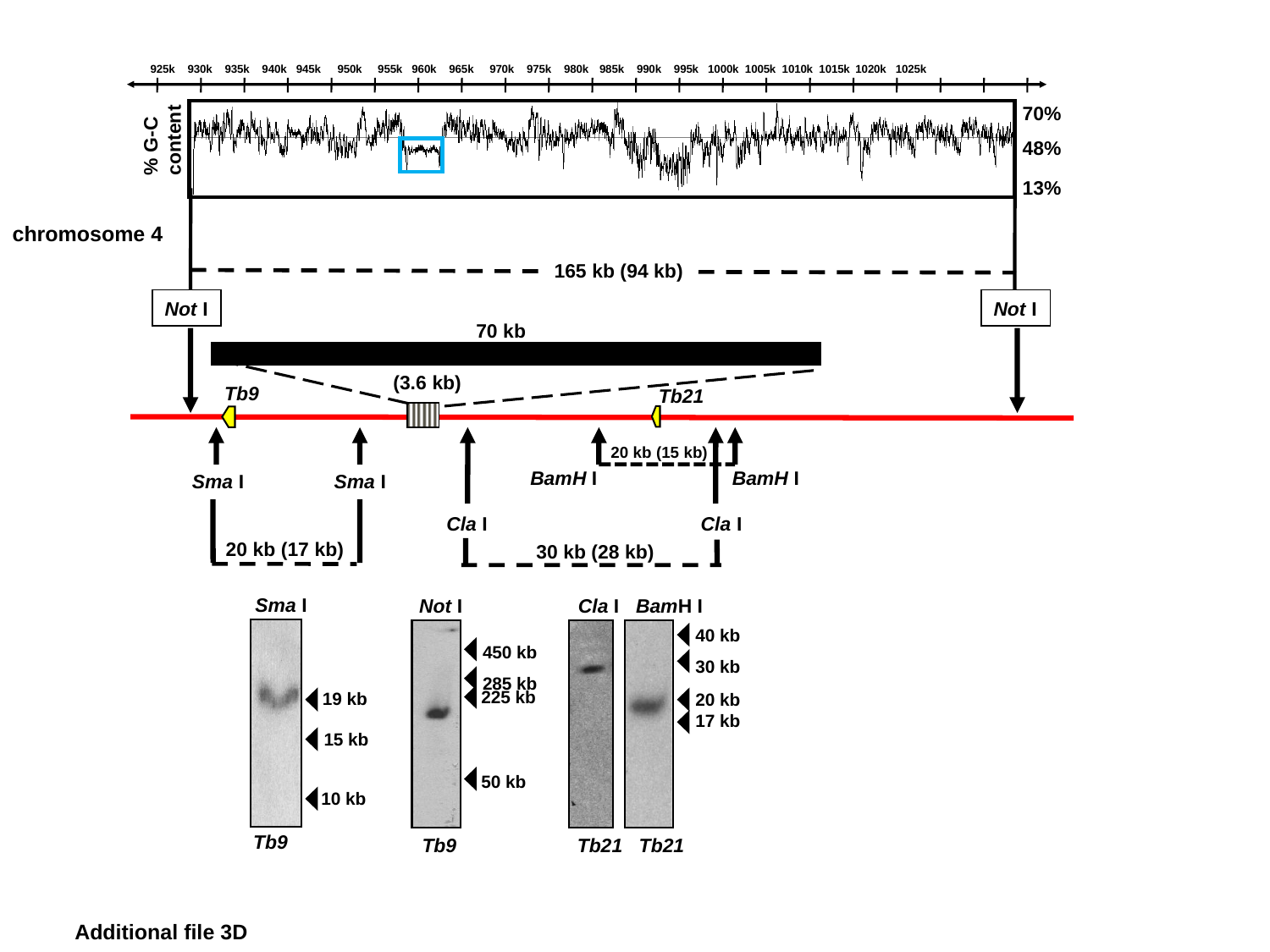

925k 930k 935k 940k 945k 950k 955k 960k 965k 970k 975k 980k 985k 990k 995k 1000k 1005k 1010k 1015k 1020k 1025k
70%
48%
% G-C
content
13%
chromosome 4
165 kb (94 kb)
Not I
Not I
70 kb
(3.6 kb)
Tb9
Tb21
20 kb (15 kb)
BamH I
BamH I
Sma I
 Sma I
Cla I
 Cla I
20 kb (17 kb)
30 kb (28 kb)
Sma I
 Not I Cla I BamH I
40 kb
30 kb
450 kb
285 kb
20 kb
17 kb
225 kb
50 kb
19 kb
15 kb
10 kb
Tb9
 Tb9 Tb21 Tb21
Additional file 3D

## Slide 5
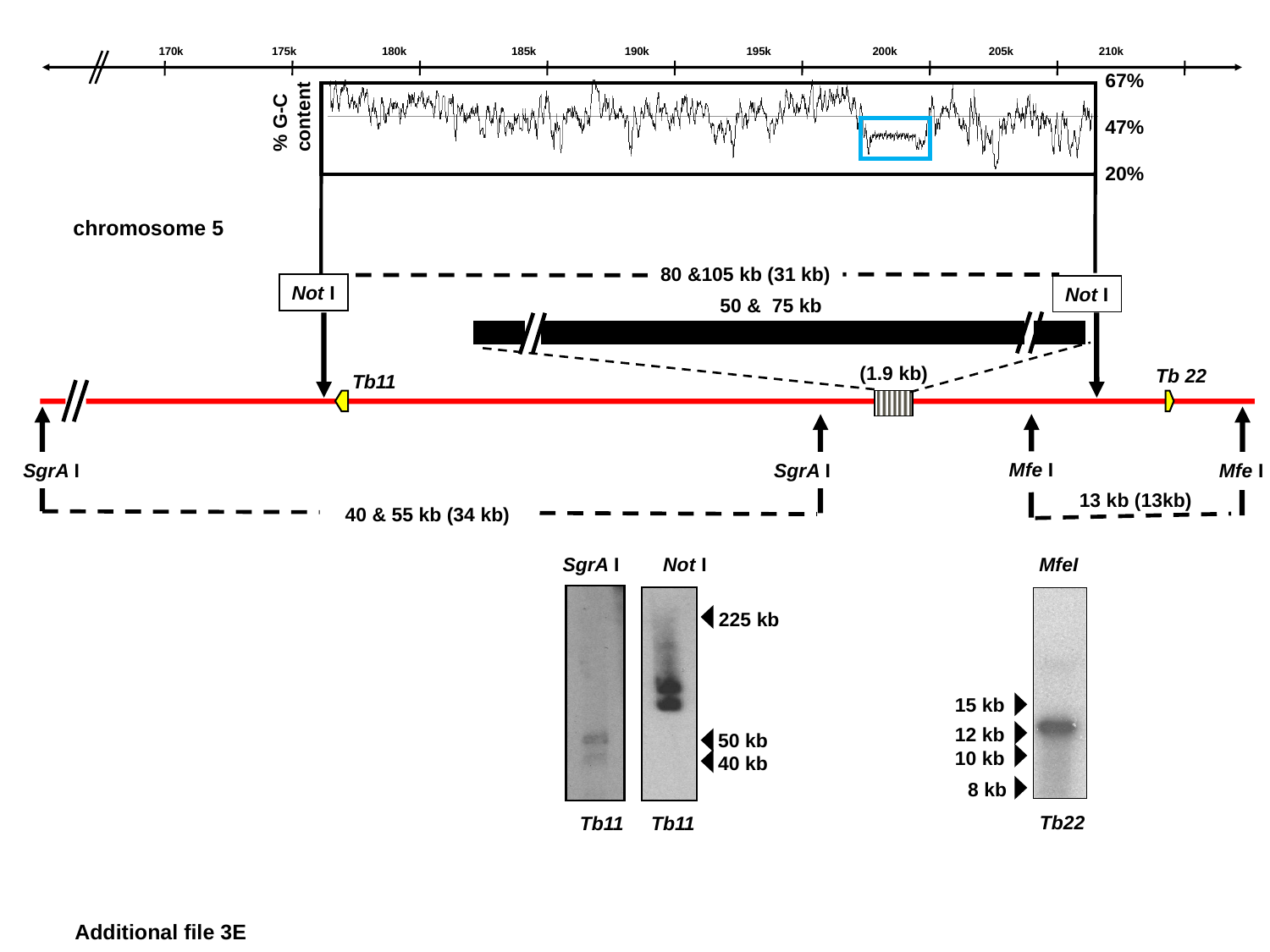

170k 175k 180k 185k 190k 195k 200k 205k 210k
67%
47%
20%
% G-C
content
chromosome 5
80 &105 kb (31 kb)
Not I
Not I
50 & 75 kb
(1.9 kb)
Tb 22
Tb11
Mfe I
SgrA I
SgrA I
Mfe I
13 kb (13kb)
40 & 55 kb (34 kb)
SgrA I Not I
MfeI
225 kb
15 kb
12 kb
10 kb
50 kb
40 kb
8 kb
Tb22
Tb11 Tb11
Additional file 3E

## Slide 6
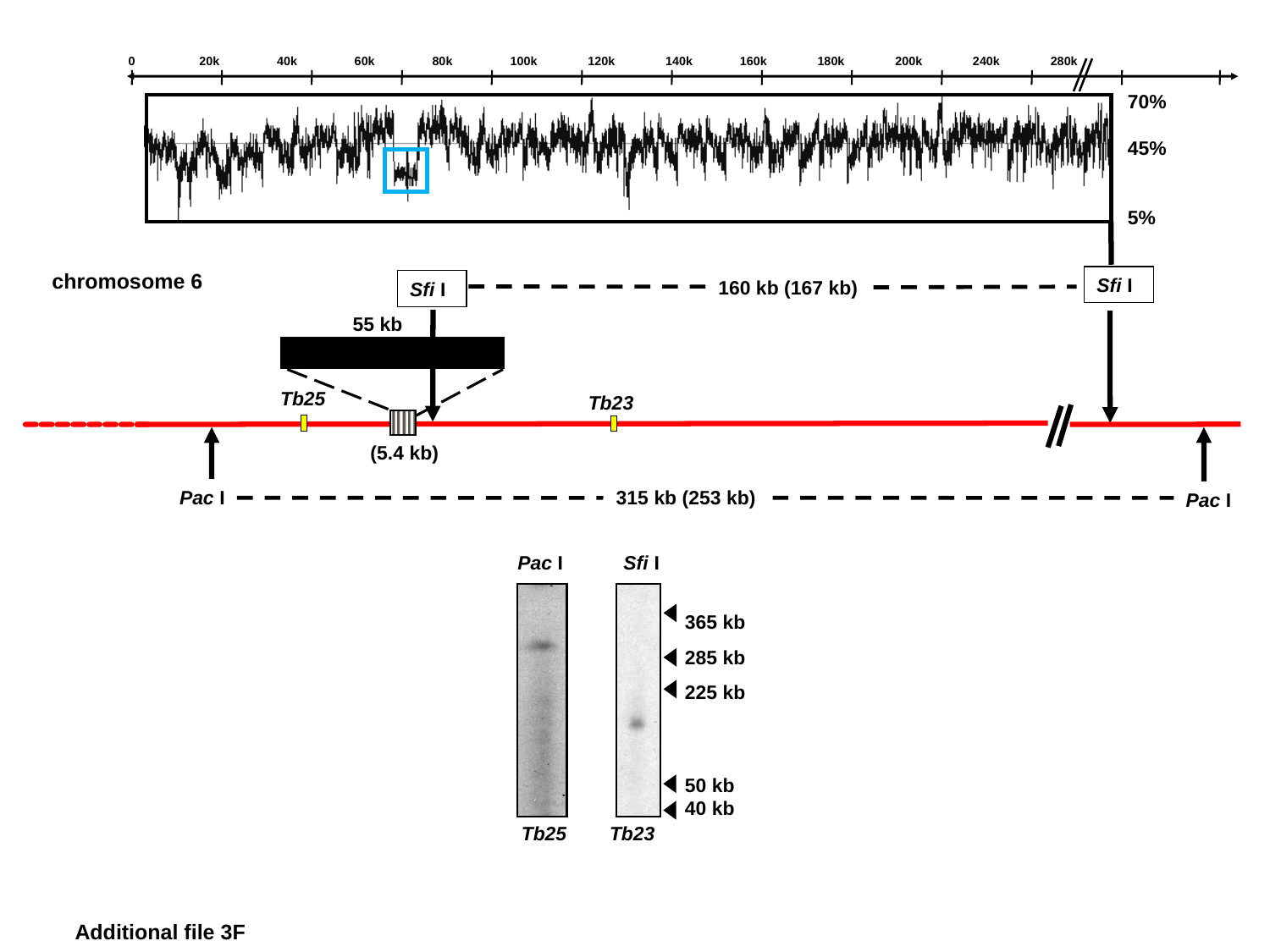

0 20k 40k 60k 80k 100k 120k 140k 160k 180k 200k 240k 280k
70%
45%
5%
chromosome 6
Sfi I
160 kb (167 kb)
Sfi I
55 kb
Tb25
Tb23
 (5.4 kb)
Pac I
315 kb (253 kb)
Pac I
Pac I Sfi I
365 kb
285 kb
225 kb
50 kb
40 kb
Tb25
Tb23
Additional file 3F

## Slide 7
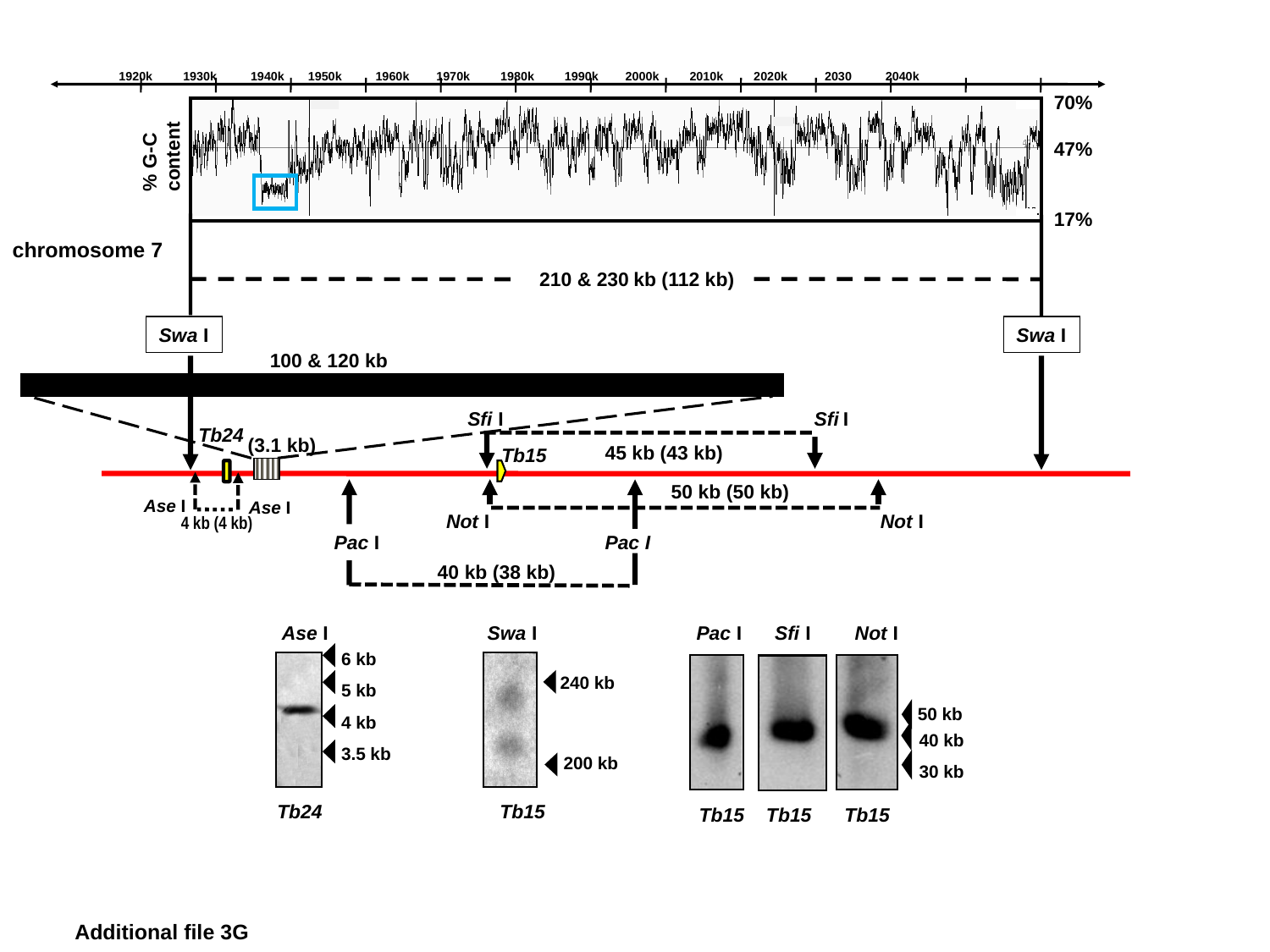

1920k 1930k 1940k 1950k 1960k 1970k 1980k 1990k 2000k 2010k 2020k 2030 2040k
70%
47%
17%
% G-C
content
chromosome 7
210 & 230 kb (112 kb)
Swa I
Swa I
100 & 120 kb
Sfi I
Sfi I
 Tb24
(3.1 kb)
45 kb (43 kb)
Tb15
50 kb (50 kb)
Ase I
Ase I
Not I
Not I
4 kb (4 kb)
Pac I
Pac I
40 kb (38 kb)
Ase I
Swa I
Pac I Sfi I Not I
6 kb
5 kb
4 kb
3.5 kb
240 kb
50 kb
40 kb
30 kb
200 kb
Tb24
Tb15
Tb15 Tb15 Tb15
Additional file 3G

## Slide 8
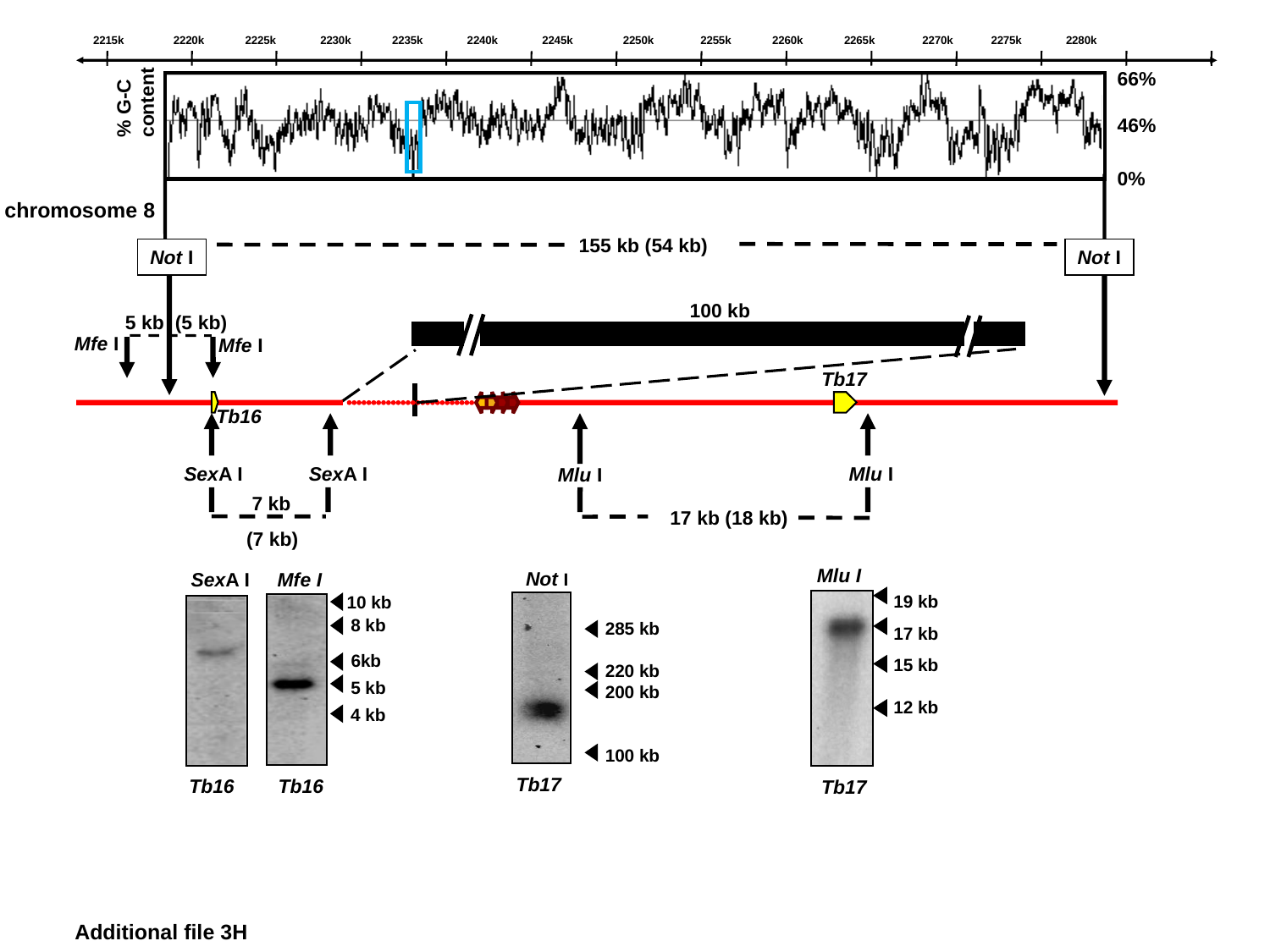

2215k 2220k 2225k 2230k 2235k 2240k 2245k 2250k 2255k 2260k 2265k 2270k 2275k 2280k
% G-C
content
66%
46%
0%
chromosome 8
155 kb (54 kb)
Not I
Not I
100 kb
5 kb (5 kb)
Mfe I
Mfe I
Tb17
Tb16
SexA I
SexA I
Mlu I
Mlu I
 7 kb
(7 kb)
17 kb (18 kb)
Mlu I
Not I
SexA I Mfe I
19 kb
17 kb
15 kb
12 kb
10 kb
8 kb
5 kb
285 kb
220 kb
200 kb
100 kb
6kb
4 kb
Tb17
Tb16 Tb16
Tb17
Additional file 3H
